# Influenza A virus inhibits TET2 expression by endoribonuclease PA-X to attenuate type I interferon signaling and promote viral replication

**DOI:** 10.1371/journal.ppat.1011550

**Published:** 2023-07-27

**Authors:** Yixiang Hu, Xinru Chen, Yuehuan Ling, Kun Zhou, Meiqing Han, Xingbo Wang, Min Yue, Yan Li

**Affiliations:** 1 Department of Veterinary Medicine & Institute of Preventive Veterinary Sciences, Zhejiang University College of Animal Sciences, Hangzhou, Zhejiang, China; 2 Hainan Institute of Zhejiang University, Sanya, Hainan, China; 3 Zhejiang Provincial Key Laboratory of Preventive Veterinary Medicine, Hangzhou, Zhejiang, China; 4 State Key Laboratory for Diagnosis and Treatment of Infectious Diseases, National Clinical Research Center for Infectious Diseases, National Medical Center for Infectious Diseases, The First Affiliated Hospital, Zhejiang University School of Medicine, Hangzhou, Zhejiang, China; University of Wisconsin-Madison, UNITED STATES

## Abstract

Influenza A virus (IAV) expresses several accessory proteins to limit host anti-viral restriction factors to facilitate viral replication. The Ten-Eleven Translocation 2 (TET2) is a methylcytosine dioxygenase that promotes DNA demethylation by catalyzing the oxidation of 5-methylcytosine (5mC) into 5-hydroxymethylcytosine (5hmC), which plays a vital role in hematopoiesis and immunity. Here we report that TET2 is a host restriction factor that limits IAV replication. But IAV endoribonuclease PA-X is able to remove the replication restriction by binding to *TET2* mRNA and driving *TET2* mRNA degradation to reduce TET2 expression during infection. Genetic inactivation of TET2 markedly enhances IAV replication *in vitro* and *in vivo*. Mechanistically, we found that TET2 regulates demethylation and transcription of *STAT1* and some interferon-stimulated genes (ISGs), including *ISG15*, *ISG20*, and *IFIT5*, so the loss of TET2 greatly impairs type I Interferon signaling. Furthermore, we confirmed that TET2-mediated demethylation of the *STAT1* gene is critical for interferon anti-viral activity. Our study demonstrates that the host TET2 is essential to the innate immune response against IAV infection.

## Author summary

Influenza A virus (IAV) severely risks public health and economic stability. The endoribonuclease PA-X of IAV can limit host gene expression to facilitate viral replication. The Ten-Eleven Translocation 2 (TET2)-mediated DNA demethylation, which promotes gene transcription, is essential for host hematopoiesis and immunity. Here, we report that host *TET2* mRNA can be targeted by PA-X protein for degradation, resulting in reduced TET2 expression during IAV infection. Notably, loss of TET2 markedly enhances IAV replication. Our study further confirmed that TET2 is responsible for the demethylation and expression of *STAT1*, *ISG15*, *ISG20*, and *IFIT5* genes, which belong to type I interferon signaling and contribute to anti-viral immune response. Additionally, we identified that STAT1 is a critical factor in response to TET2 in the anti-IAV signaling. Taken together, we demonstrated that IAV inhibits TET2 expression by PA-X to reduce *STAT1* and ISG expression, leading to immune escape and enhanced viral replication. Our research can provide a novel target for therapeutics to inhibit IAV infection and reduce pathology.

## Introduction

The influenza A virus (IAV) is an enveloped virus with a genome composed of eight segmented negative-stranded RNAs (PB2, PB1, PA, HA, NA, NP, M, and NS) belonging to the family *Orthomyxoviridae* [1]. IAV causes mild to severe respiratory disease and has brought about four pandemics in the past 100 years, i.e., 1918 H1N1, 1957 H2N2, 1968 H3N2, and 2009 H1N1. Many animal species, including poultry, wildfowl, and domestic and wild mammals, can be infected by IAV [2]. IAV spillover from animal to human sporadically occurred, resulting in severe public health risks and tremendous economic loss.

IAV mainly targets the epithelial cells in the respiratory tract and infects other types of cells, such as endothelial cells, natural killer cells, macrophages and dendritic cells [3, 4]. Upon IAV infection, the host pattern recognition receptors (PRRs), including Toll-like receptor (TLR)3/7/8 and retinoic acid-inducible gene-I (RIG-I), quickly recognize viral RNAs and intermediate RNAs, respectively. Following intracellular sensing by PRRs, activated IFN regulatory factor (IRF)3/7 and NF-κB induce the transcription of type I interferon (IFN) in the nucleus. Subsequently, secreted type I IFN binds to IFN-α/β receptor complex (IFNAR), transferring signals through Janus kinase (JAK)-signal transducer and activator of transcription (STAT1) pathway, which phosphorylates STAT1 and STAT2 [5,6]. Notably, STAT1 activation requires the phosphorylation of Y701 and S727 and the methylation of amino-terminal arginine [7,8]. IAV PB2 protein inhibits STAT1 phosphorylation by targeting JAK1 for ubiquitination and degradation [9]; or by inducing host SOCS1 and SOCS3 expression, the suppressor of the JAK-STAT pathway, by viral RNA and NS1 protein [10,11]. However, little is known about the regulation of STAT1 expression by IAV. Eventually, the phosphorylated STAT1 and STAT2, and IRF9, form together the interferon-stimulated gene factor 3 (ISGF3), which translocates to the nucleus to activate the transcription of hundreds of IFN-stimulated genes (ISGs), some of which are critical effectors against viral infection [5,6]. For example, ISG15 can disrupt the functions of the viral NS1 protein by ISGylation [12,13], ISG20 is an exonuclease targeting viral single-stranded RNA [14,15], and IFIT family proteins can directly bind viral 5’-triphosphate RNA to hinder viral RNA translation [16,17].

IAV expresses multiple virulence factors to suppress host gene expression during infection, known as “host shutoff” [18,19], to create an intracellular environment suitable for viral replication. The endoribonuclease PA-X is translated from the polymerase acidic protein (PA) mRNA by +1 ribosomal frameshifting. PA-X and PA share the identical 191 amino acids in the amino-terminal ribonuclease (RNase) domain, and PA-X (aa) has a unique carboxy-terminal domain of 41 or 61 aa known as the X-open reading frame (X-ORF) [20–22]. The RNase domain is responsible for mRNA degradation, and the X-ORF domain plays an essential role in PA-X nuclear localization and functionality [23–26]. PA-X is the primary driving force in general host shutoff while preferentially downregulating host spliced pol II transcripts. The X-ORF domain can interact with the proteins involved in the host mRNA processing [27–29]. Previous studies have shown that PA-X in some IAV strains disrupts host immune responses by downregulating interferons (IFNs) and pro-inflammatory cytokines, including TNFα, IL-1β, and IL-6, to protect the host and reduce mortality [30,31]. Yet, little is known about the consequences of PA-X targeting other host genes.

The methylcytosine dioxygenase Ten-Eleven Translocation 2 (TET2) plays a crucial role in DNA demethylation as it catalyzes the conversion of 5-methylcytosine (5mC) into 5-hydroxymethylcytosine (5hmC) [32–35]. It is also an essential regulator for normal hematopoiesis, especially myelopoiesis [36,37]. Recently, numerous studies revealed that TET2 plays a crucial role in host immune responses and inflammation. One study in plasmacytoid dendritic cells (pDCs) shows that CXXC5 could recruit Tet2 to maintain hypomethylation of CpG islands in the promoter of *Irf7*, eliciting type I IFN production for anti-viral immune response activation [38]. Tet2 could also mediate the oxidation of 5mC on the 3’-UTR of *Socs3* mRNA in innate immune cells, promoting the Adar1-mediated degradation of *Socs3* mRNA and facilitating infection-induced myelopoiesis [39]. Besides the oxidase activity-dependent manner, Tet2 could be recruited by IκBζ to *Il-6* promoter and, in turn, recruit Hdac1/2 to induce histone deacetylation, thus suppressing *IL-6* transcription during the resolution stage of inflammation [40,41]. The repression of TET2 on IL-6 was further verified in a study about HIV immune escape, in which it reveals that HIV Vpr protein targets TET2 for degradation by the proteasome to sustain IL-6 expression to enhance viral replication [42]. And a subsequent study found that the Vpr-TET2 axis also enhances HIV-1 replication in macrophages by reducing TET2-mediated demethylation and expression of host interferon-induced transmembrane protein 3 (IFITM3) [43]. These studies suggested that HIV could modulate TET2-dependent immune response and inflammation. Besides, TET2 has also been found to intervene in the infection of other viruses, including Epstein-Barr virus (EBV) [44,45] and swine acute diarrhea syndrome coronavirus (SADS-CoV) [46]. To date, whether IAV infection modifies TET2 expression and the role of TET2 in the immune response during IAV infection remains unclear.

Here, we investigated the effects and mechanisms of TET2 on IAV infection in TET2 deletion cells *in vitro*. We also applied a mouse model to verify the role of Tet2 on IAV infection *in vivo*. We found that IAV infection inhibited TET2 expression via the endonuclease activity of PA-X. This led to the suppressed expression of IFN signaling genes regulated by TET2 demethylation, including STAT1 and some ISGs, thus ultimately promoting IAV replication. In particular, we confirmed that TET2-mediated demethylation of the *STAT1* gene is critical for interferon anti-viral activity. Together, our study demonstrated that TET2, acting as a positive regulator of the IFN signaling cascade through demethylation, can facilitate anti-viral defense and attenuate IAV infection.

## Results

### IAV infection reduces TET2 expression

**Fig 1:**
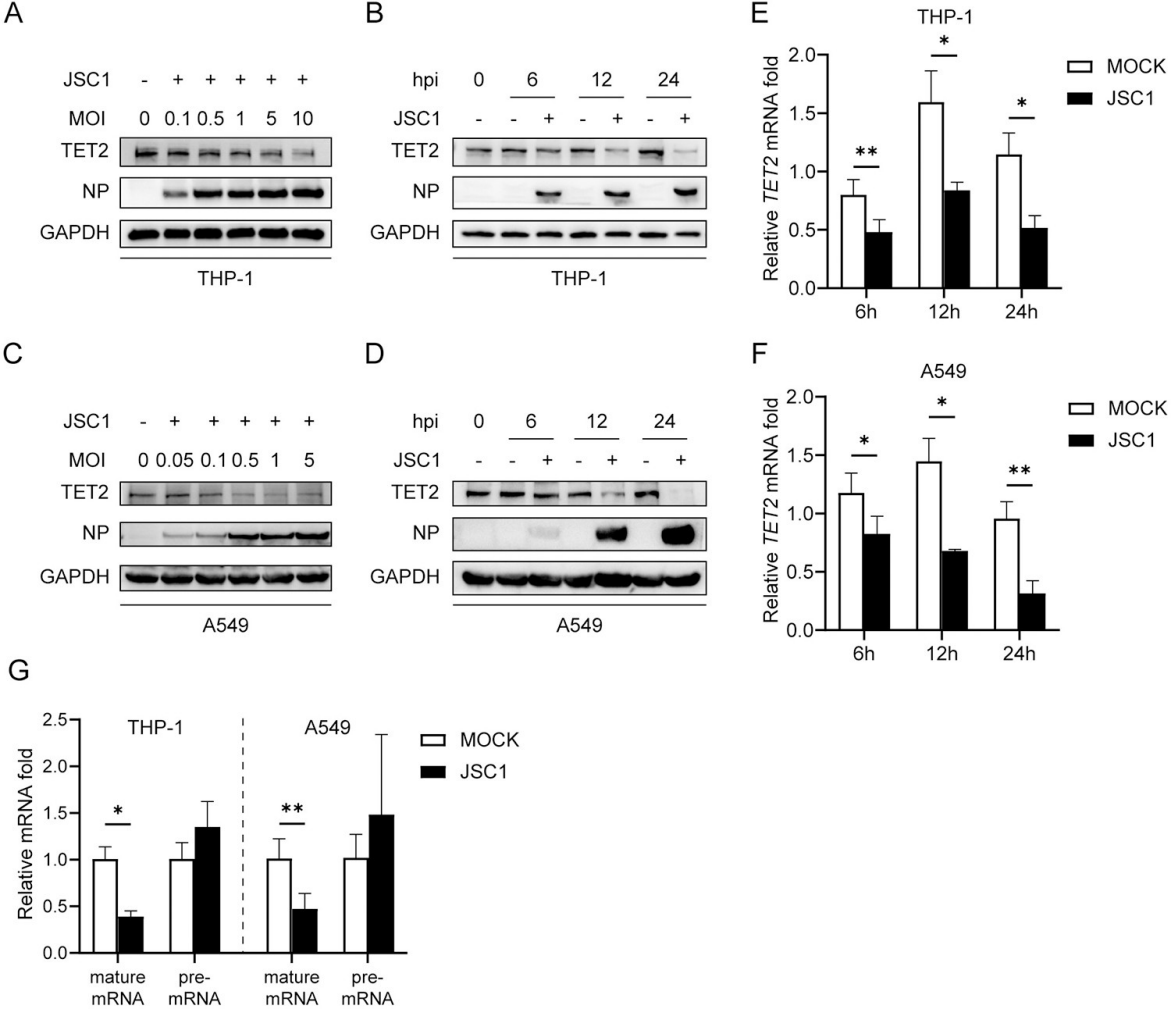
TET2 expression is reduced in H9N2 JSC1 infected THP-1 cells and A549 cells. THP-1 cells (A) or A549 cells (C) were infected with JSC1 at indicated MOIs and harvested at 9 hours post-infection (hpi). THP-1 cells (B and E) or A549 cells (D and F) were infected with JSC1 at an MOI of 1 and harvested at 0, 6, 12 and 24 hpi. (A-D) Protein levels of TET2, viral NP, and GAPDH were analyzed by Western Blot. (E and F) *TET2* mRNA and *18S rRNA* levels were detected by RT-qPCR. The data are expressed as fold changes in *TET2* mRNA levels at 6, 12, and 24 hpi relative to 0 hpi control. (G) THP-1 cells or A549 cells were infected with JSC1 at an MOI of 1 and harvested at 12 hpi. *18S rRNA*, *TET2* mRNA and *TET2* pre-mRNA levels were detected by RT-qPCR. The data are expressed as fold changes relative to the mock infections. Error bars represent ± SD for triplicate experiments. Statistical analysis was performed using Student’s t test. *p < 0.05, **p < 0.01, ***p < 0.001.

Firstly, we examined the TET2 expression in cells after IAV infection. Human THP-1 cells were infected with influenza virus A/swine/Jiangsu/C1/2008 (H9N2) (JSC1) at an MOI of 0.1, 0.5, 1, 5, and 10, and the cells were collected after 9 hours post-infection (hpi). As the MOI increased, the TET2 expression decreased accordingly (Fig 1A). Then we tested the dynamic change of TET2 in THP-1 cells with H9N2 JSC1 infected at an MOI of 1, and we observed the TET2 protein levels decreased gradually in the infected cells over time, with a ∼60% decline as compared with uninfected cells at 24 hpi (Fig 1B). We also examined TET2 expression upon IAV infection in the A549 human alveolar epithelial cell line. Consistently, TET2 protein level decreased in infected A549 cells in the infectious dose- and time-dependent manner (Fig 1C and 1D). These data indicate that IAV infection decreases TET2 expression. To test whether the decreased TET2 protein level was due to the decreased mRNA level, we detected the mature mRNA of *TET2* and found a significant decrease of *TET2* mature mRNA level at 6, 12, and 24 hpi (Fig 1E and 1F). The *TET2* gene locates in the human chromosome 4 and contains 11 exons [32]. After transcription from the genome DNA, the pre-mRNA transcript undergoes post-transcriptional modifications, such as RNA splicing, to give rise to the functional mature mRNA [47]. To further examine whether a decrease of *TET2* mRNA happened during or after gene transcription, we examined the level of pre-mRNA transcript by qPCR with the primers crossing the adjacent exon and intron. As shown in Fig 1G, the level of *TET2* pre-mRNA did not change after IAV infection, suggesting that the down-regulation of *TET2* mRNA by IAV infection occurs at the post-transcriptional modification stage rather than the transcriptional stage. These results indicate that IAV infection inhibits TET2 protein expression through pre-mRNA post-transcriptional regulation.

### PA-X inhibits TET2 expression by degradation of *TET2* mRNA

**Fig 2:**
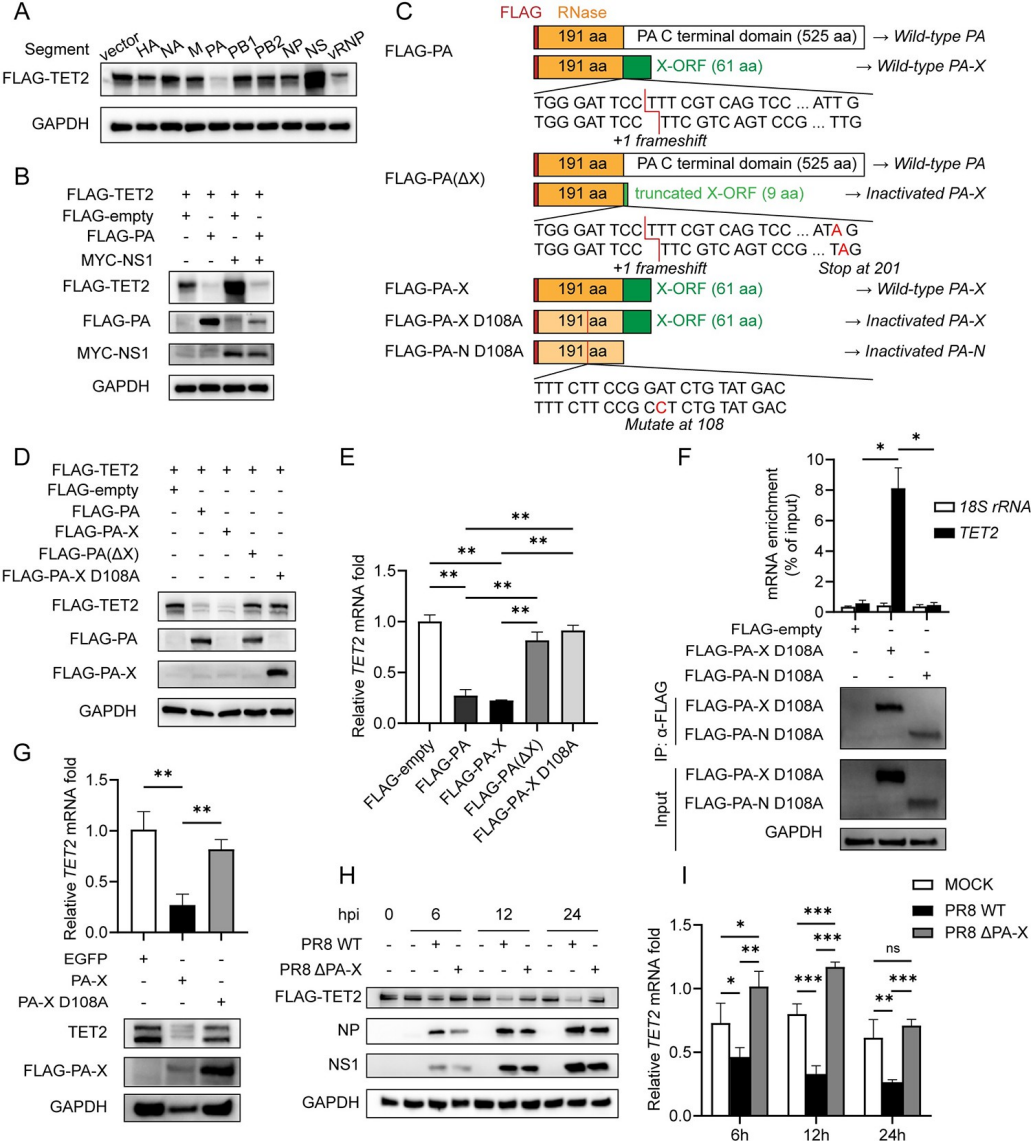
PA-X targets *TET2* mRNA for degradation with dependence on the endoribonuclease activity. (A, B and D) HEK293T cells were transfected with the indicated plasmids, and whole-cell extracts were prepared at 24 hours post-transfection (hpt), followed by immunoblotting with indicated antibodies. (C) Diagrams of expressing proteins in indicated plasmids are shown. (E) HEK293T cells were transfected with the indicated plasmids and harvested at 24 hpt. *TET2* mRNA and *18S rRNA* levels were detected by RT-qPCR. The data are expressed as fold changes relative to the FLAG-empty control. (F) RNA-binding protein immunoprecipitation (RIP) was performed with anti-FLAG M2 agarose beads in HEK293T cells transfected with FLAG-PA-X D108A, FLAG-PA-N D108A or FLAG-empty for 24 hours, followed by immunoblotting to verify the efficiency of immunoprecipitation, and by RT-qPCR to measure the *TET2* mRNA and *18S rRNA* levels. RNA enrichment was calculated by normalization of each IP fractions’ Ct to the input fraction’ Ct in the same qPCR assay (ΔCt). (G) RNA and protein samples were collected from THP-1 cells expressing doxycycline-inducible FLAG-PA-X, FLAG-PA-X D108A, or EGFP 18 hours after the supplement of doxycycline, followed by immunoblotting analysis and RT-qPCR detection. The RT-qPCR data are expressed as fold changes relative to the EGFP control. (H and I) Protein and RNA samples were collected from THP-1 cells infected with either PR8 WT or PR8 ΔPA-X at an MOI of 1, followed by immunoblotting analysis (H) and RT-qPCR detection (I). Error bars represent ± SD for triplicate experiments. Statistical analysis was performed using Student’s t test or ANOVA method. *p < 0.05, **p < 0.01, ***p < 0.001.

To explore the mechanism of IAV-mediated TET2 expression reduction, we transfected HEK293T cells with plasmids containing each viral segment of H1N1 PR8 (PB1, PB2, PA, NP, HA, NA, M, and NS) or with plasmids containing genes encoding ribonucleoprotein (RNP) complex proteins (PB1, PB2, PA, and NP), and followed by TET2 protein detection by Western Blot at 24 hours post-transfection (hpt). The results showed that the expression of TET2 was strongly inhibited by the PA segment and the RNP complex carrying the PA segment (Fig 2A). Similar to what Salvatore et al. observed that NS1 protein enhanced protein expression of transfected plasmid by translational improvement [48,49], the expression of TET2 was up-regulated by co-transfection with the NS segment (Fig 2A). We then transfected the plasmids expressing PA or NS1 protein separately or together, and found the plasmid expressing PA could inhibit the expression of TET2 and counteract the up-regulation of TET2 by NS1 (Fig 2B). Hence, we speculated that the PA segment was dominant in regulating TET2 expression among IAV segments.

In addition to the protein PA, the PA segment also encodes a shorter protein, PA-X, with more robust RNA endonuclease activity by +1 ribosomal frameshifting during IAV infection [20–22]. To investigate whether protein PA or PA-X participates in the down-regulation of TET2, we introduced a nonsense mutation L201Stop in PA-X to generate PA(ΔX), which truncated the X-ORF after nine amino acids (aa) (Fig 2C). It would greatly lose its host shutoff activity [23,29] but still keep the amino acid sequence of PA. In addition, we also constructed plasmids expressing PA-X and its catalytically inactive mutant PA-X D108A [29,50,51] (Fig 2C). The plasmids expressing TET2 were co-transfected with plasmids expressing PA, PA-X, PA(ΔX), PA-X D108A, or empty vector into HEK293T cells. After 24 hours, the cells were taken down to examine the TET2 protein levels by Western Blot and *TET2* mRNA levels by RT-qPCR. The results showed that the plasmids expressing wild-type PA-X reduced TET2 protein and mRNA levels, but the plasmids expressing only the PA or mutant PA-X did not (Fig 2D and 2E). These results suggest that PA-X, but not PA, plays a significant role in TET2 expression inhibition with dependence on the endonuclease activity. Lv et al. have demonstrated that HIV-1 Vpr targets TET2 for degradation during HIV infection [42]. We also examined whether PA-X could induce TET2 protein degradation. But the reduction of TET2 protein by PA-X cannot be counteracted by treating cells with inhibitors of the proteasome, calpain, caspase, and lysosome (S1A Fig), the four known degradation pathways of TET2 protein [42], and it indicated that PA-X could not induce TET2 protein degradation.

We next examined the interaction of PA-X with *TET2* mRNA by performing RNA immunoprecipitation (RIP) using an anti-FLAG antibody that binds to FLAG-PA-X D108A. The D108A mutation abolishes the endonuclease activity of PA-X, so the cellular *TET2* mRNA would be retained when the FLAG-PA-X D108A plasmids are transfected. We also constructed a plasmid FLAG-PA-N D108A. It expresses inactivated N-terminal endonuclease domain of PA-X but not the X-ORF domain (Fig 2C). We found that *TET2* mRNA can be efficiently pulled down in FLAG-PA-X D108A transfected cells but not in FLAG-PA-N D108A transfected cells (Fig 2F), indicating PA-X physically interact with *TET2* mRNA by X-ORF domain. We further examined the changes in RNA levels after ectopic PA-X expression via doxycycline (DOX)-inducible expression system in both THP-1 and A549 cells. We found that TET2 protein and mRNA levels in wild-type PA-X expressing cells dramatically decreased after 18 hours of DOX treatment (Figs 2G and S1B), but not in PA-X D108A (inactivated PA-X) expressing cells, which confirmed again that PA-X could inhibit the expression of TET2 by its RNA endonuclease activity.

To further evaluate the role of IAV PA-X in downregulating *TET2* expression during infection, we rescued the PA-X-deficient H1N1 PR8 virus (PR8 ΔPA-X) by reverse genetics (S1C Fig). The deficiency of PA-X results in slightly reduced viral replication in MDCK cells, as measured by TCID_50_ assay of viral load in the supernatant (S1D Fig). Consistent with the results from the plasmids transfection, PR8 ΔPA-X infection did not reduce TET2 protein and mRNA levels (Fig 2H and 2I). Besides, *TET2* mRNA levels slightly elevated upon PR8 ΔPA-X infection (Fig 2I), suggesting TET2 transcription could be stimulated by IAV infection, but PA-X degraded the transcripts. Taken together, IAV can downregulate *TET2* mRNA level through PA-X’s RNA endonuclease activity, thereby inhibiting TET2 protein expression.

### Loss of TET2 enhances IAV replication

**Fig 3:**
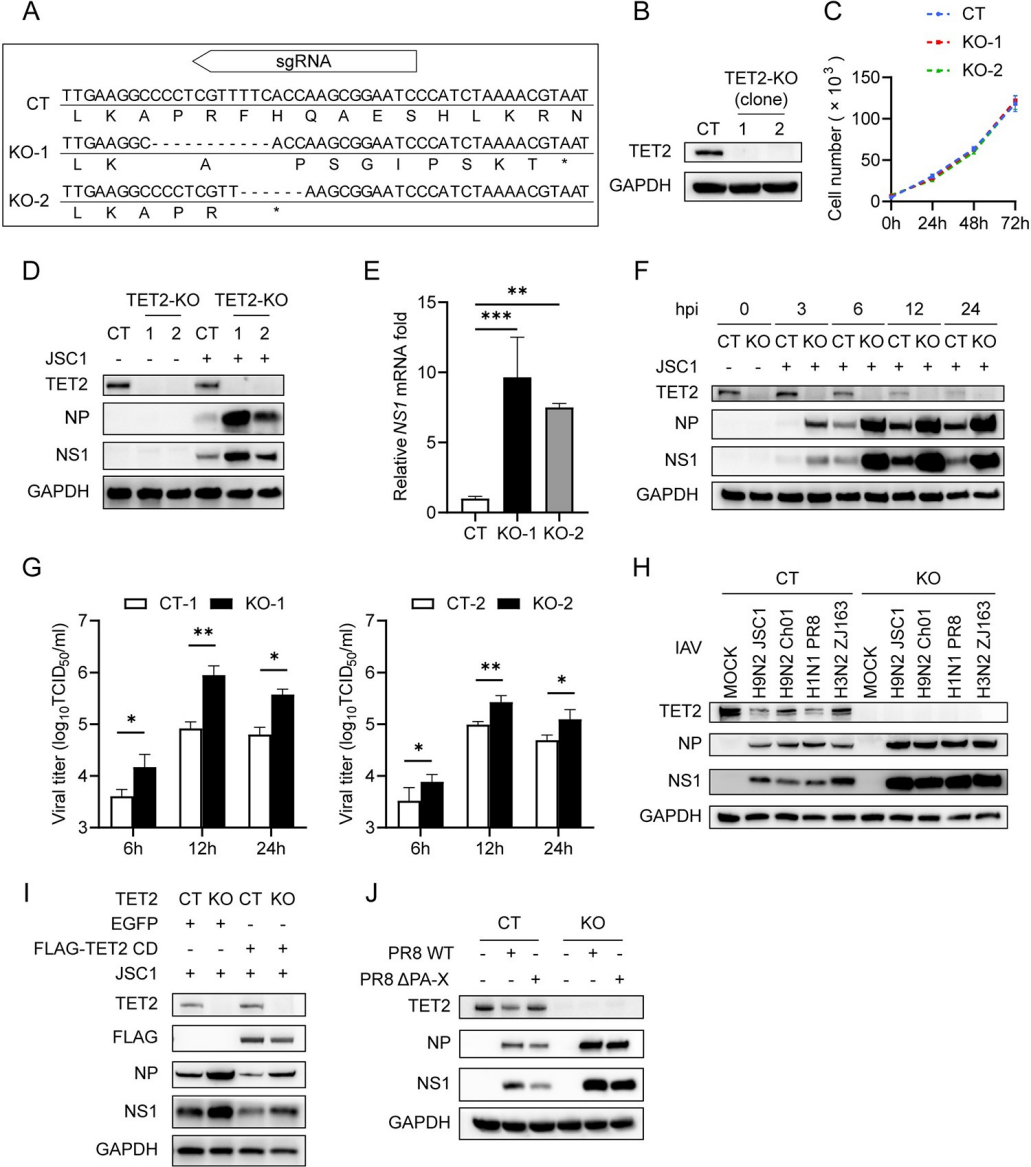
Loss of TET2 enhances IAV replication. CRISPR-Cas9-mediated TET2 knockout (KO) and non-targeting gRNA control (CT) THP-1 cells were established. (A) DNA sequencing results of sgRNA-targeted TET2 genomic region in two isolated TET2-KO single cell clones (KO-1 and KO-2) and CT THP-1 cells are shown. Hyphen (-) represents missing DNA bases, and asterisk (*) indicates the termination codon (TAA). (B) Western Blot confirmation of TET2 knockout in THP-1 cells. (C) CT, KO-1 and KO-2 THP-1 cells were seeded in the 96-well plate at equal amount cells (8 × 10^3^). Cell numbers were detected at indicated hours using Cell Counting Kit-8 (CCK-8) reagents. (D and E) CT, KO-1 and KO-2 THP-1 cells were infected with H9N2 JSC1 at an MOI of 1 and harvested at 6 hpi. (D) Protein levels of TET2, viral NP, viral NS1 and GAPDH were analyzed by Western Blot. (E) mRNA levels of viral NS1 were detected by RT-qPCR, and the data are expressed as fold changes relative to the control group. (F) Control and TET2-KO THP-1 cells were infected with JSC1 at an MOI of 1 and harvested at 0, 3, 6, 12 and 24 hours, followed by immunoblotting with indicated antibodies. (G) Control and TET2-KO THP-1 cells were infected with JSC1 at an MOI of 1, and the culture supernatants were collected at 6, 12 and 24 hpi for viral titration by TCID_50_ assay. (H) Control and TET2-KO THP-1 cells were infected with H9N2 JSC1, H9N2 Ch01, H1N1 PR8 or H3N2 ZJ163 at an MOI of 1 and harvested at 6 hpi, followed by immunoblotting with indicated antibodies. (I) Control and TET2-KO THP-1 cells transduced with lentiviral vectors expressing EGFP or FLAG-TET2 CD domain were infected with JSC1 at an MOI of 1 and harvested at 6 hpi, followed by immunoblotting with indicated antibodies. (J) Control and TET2-KO THP-1 cells were infected with either PR8 WT or PR8 ΔPA-X at an MOI of 1 and harvested at 6 hpi, followed by immunoblotting with indicated antibodies. Error bars represent ± SD for triplicate experiments. Statistical analysis was performed using Student’s t test or ANOVA method. *p < 0.05, **p < 0.01, ***p < 0.001. CT: control cells, KO: TET2 knockout cells.

To study the effect of TET2 on IAV infection, we generated TET2 knockout (TET2-KO) THP-1 cells using the CRISPR-Cas9 system. Two clones (clone 1 and clone 2) of cells with TET2 deletion were screened out by Sanger sequencing, and the TET2 deficiency was confirmed by Western Blot (Fig 3A and 3B). The cell proliferation assay showed that TET2 deletion did not affect THP-1 cell proliferation (Fig 3C). We examined the virus growth in the TET2-KO and control cells. RT-qPCR analysis of intracellular NS1 transcription at 6 hpi showed a significant ~10-fold (*p* < 0.001) or ~8-fold (*p* < 0.01) increase in TET2-KO-1 or TET2-KO-2 cells (Fig 3E). Western Blot analysis of viral NP and NS1 expression showed 2~8-fold increases at 3, 6, 12, and 24 hpi in the TET2-KO cells (Fig 3D and 3F). TCID_50_ assay of viral load in the supernatant demonstrated a significant ~10-fold (*p* < 0.01) increase in TET2-KO-1 cells (Fig 3G). To avoid the inherent heterogenicity of the parental cell population, we compared viral growth in TET2-KO-2 cells and other control clone cells. Consistent with clone 1, TET2 deletion in clone 2 also promoted the production of progeny viruses (Fig 3G). These results indicate the loss of TET2 facilitate H9N2 JSC1 replication in THP-1 cells.

To further confirm whether TET2 has broad anti-IAV activity, we infected THP-1 cells with several other IAV strains, including H1N1 PR8, H3N2 ZJ163, and H9N2 Ch01 at an MOI of 1 for 6 hours. Consistent with H9N2 JSC1, the NP and NS1 expressions of the other IAV strains were also elevated in TET2-KO THP-1 cells (Fig 3H). Moreover, the expression of TET2 protein was also decreased by infection of different subtypes of IAV (Fig 3H), hinting at a conserved effect of IAV infection on TET2 expression. To verify the anti-IAV effect of TET2 and exclude the off-target impact related to the CRISPR-Cas9 system, we ectopically expressed the catalytic center of TET2, cysteine-rich dioxygenase domain (CD domain) [33,35,52] in TET2-KO THP-1 cells and control THP-1 cells. Expressing the TET2 CD domain notably inhibited viral NP and NS1 expression in control THP-1 cells and greatly impaired the increased viral proteins in TET2-KO THP-1 cells (Fig 3I). In addition, while PA-X deficiency could reduce viral replication and protein expression (Figs S1D, and 2H, 3J), TET2 deletion still caused a sharp increase in viral protein expression regardless of viral PA-X (Fig 3J). Moreover, we established a TET2 haploinsufficiency A549 cell line using CRISPR-Cas9. Although only about half of TET2 was abolished, the NP and NS1 expression of JSC1 increased nearly 2-fold compared with that in the control A549 cells at 12 hpi (S2 Fig). These results suggest that TET2 plays an essential role in cellular anti-IAV activity, and the anti-IAV activity of TET2 relies on its catalytic center CD domain.

### Loss of TET2 impairs interferon (IFN) anti-viral activity by reducing the demethylation of IFN signaling pathway genes

**Fig 4:**
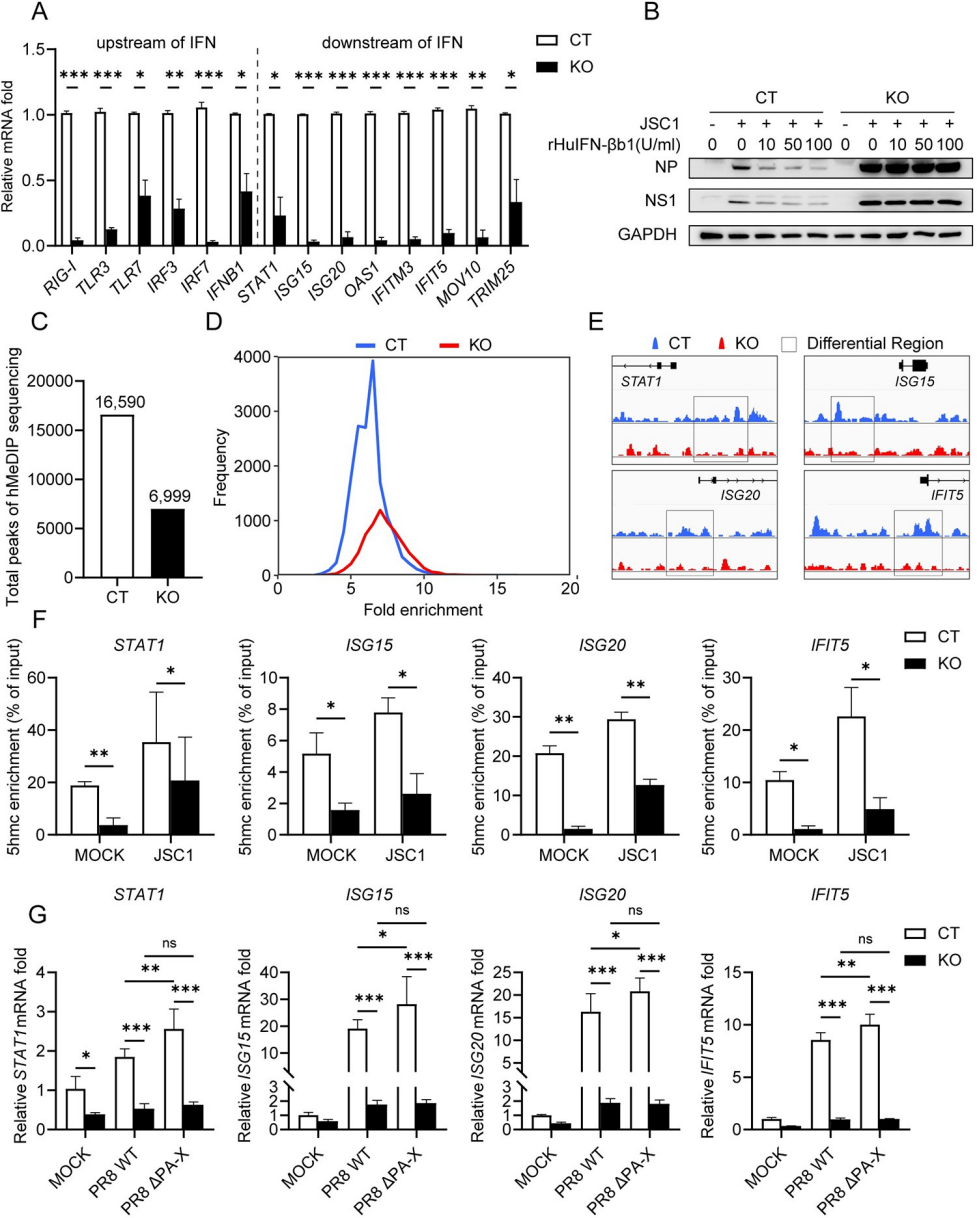
TET2 deletion attenuates the demethylation and transcription of *STAT1* and ISGs. (A) Control and TET2-KO THP-1 cells were infected with JSC1 at an MOI of 1. At 6 hpi, total RNA was extracted from cells, and *RIG-1*, *TLR3*, *TLR7*, *IRF3*, *IRF7* and *IFNB1* mRNA levels (upstream of IFN) and *STAT1*, *ISG15*, *ISG20*, *IFITM3*, *OAS1*, *IFIT5*, *MOV10* and *TRIM25* mRNA levels (downstream of IFN) were evaluated via RT-qPCR. The data are expressed as fold changes relative to the control group. (B) Control and TET2-KO THP-1 cells were treated with indicated amounts of rHuIFN-βb1 for 2 hours and then infected with JSC1 at an MOI of 1 and harvested at 6 hpi, followed by immunoblotting with indicated antibodies. (C-E) Hydroxymethylated DNA immunoprecipitation and sequencing (hMeDIP-Seq) was performed in control and TET2-KO THP-1 cells infected with JSC1 at an MOI of 3 for 6 hours. (C) Peaks were identified using the MACS algorithm (*p*-value = 1e-5). The bar chart displays the numbers of total peaks. (D) Distribution of signal value are shown, which indicates the frequency of peaks with different fold enrichments. (E) Distribution of 5hmC along promoter regions of *STAT1* (chr2 191,876,601–191,882,599), *ISG15* (chr1 945,390–951,388), *ISG20* (chr15 89,175,708–89,181,706) and *IFIT5* (chr10 91,170,072–91,176,070) genes in control (blue) and TET2-KO (red) cells are shown. The regions of differential 5hmC levels are highlighted by black squares. (F) hMeDIP assays were performed in control and TET2-KO THP-1 cells with or without JSC1 infection. 5hmC levels on the *STAT1*, *ISG15*, *ISG20* and *IFIT5* promoters were determined by qPCR. 5hmC enrichment was calculated by normalization of each IP fractions’ Ct to the input fraction’ Ct in the same qPCR assay (ΔCt). (G) Control and TET2-KO THP-1 cells were infected with either PR8 WT or PR8 ΔPA-X at an MOI of 1 and harvested at 6 hpi, and mRNA levels of *STAT1*, *ISG15*, *ISG20* and *IFIT5* were evaluated via RT-qPCR. Error bars represent ± SD for triplicate experiments. Statistical analysis was performed using Student’s t test or ANOVA method. *p < 0.05, **p < 0.01, ***p < 0.001. CT: control cells, KO: TET2 knockout cells.

The IFN pathway is a potent defense line of cellular immunity against influenza by stimulating the expression of hundreds of IFN-stimulated genes (ISGs), some of which are critical effectors limiting influenza virus replication [5,6]. Thus, we detected the mRNA levels of some crucial upstream genes in the IFN pathway and ISGs against IAV at 6 hpi, including *RIG-I*, *TLR3*, *TLR7*, *IRF3*, *IRF7*, *IFNB1*, *STAT1*, *ISG15*, *ISG20*, *IFITM3*, *OAS1*, *IFIT5*, *MOV10* and *TRIM25*. As shown in Fig 4A, the expression of these genes was reduced by 60%~97% in the TET2-KO cells, which indicated that TET2 expression is required for strong expression of IFN pathway genes. Besides, *IFNB1* was also downregulated in the TET2 haploinsufficiency A549 cell line (S3A Fig). It has been shown that TET2 could regulate IFNβ expression by demethylating IRF7 [38], suggesting decreased IFN expression might be the reason of immune deficiency in the TET2-KO cells. However, exogenous supplementation of recombinant rHuIFN-βb1 could not counteract the increased viral replication in TET2-KO cells (Fig 4B), suggesting that TET2 might target the genes downstream of IFN signaling.

TET2 oxidizes the methyl group of 5mC to 5hmC to facilitate the demethylation of CpG DNA [33–35]. To unravel which genes are regulated by TET2 upon IAV infection, we performed a hydroxymethylated DNA immunoprecipitation and high-throughput sequencing (hMeDIP-Seq) experiment to analyze the 5-hydroxymethylome in JSC1 infected control and TET2-KO THP-1 cells. There were ~2.4 times fewer total peaks of hMeDIP-Seq in TET2-KO cells than in control cells (Fig 4C and 4D), which suggested the demethylation levels in TET2-KO cells markedly decreased. We compared the 5hmC levels in the TET2-KO and control cells and found that *STAT1*, *ISG15*, *ISG20*, and *IFIT5* have lower 5hmC levels near their promoter regions in the TET2-KO cells (Fig 4E). We further verified the results of hMeDIP-Seq by hMeDIP-qPCR. As shown in Fig 4F, the 5hmC levels of *STAT1*, *ISG15*, *ISG20*, and *IFIT5* in the uninfected TET2-KO cells were only 7%~30% of those in the uninfected control cells. Upon IAV infection, the 5hmC levels of *STAT1*, *ISG15*, *ISG20*, and *IFIT5* increased, but the levels in the IAV-infected TET2-KO cells were still 22%~59% of those in IAV-infected control cells. The mRNA levels of these genes in the TET2-KO THP-1 cells were slightly reduced in the mock-infected cells, but considerably lower in the IAV-infected cells (Fig 4G). Although virus replication slightly rises in the A549 cells with TET2 haploinsufficiency, mRNA levels of these genes were also markedly downregulated at 12 hpi (S3B-S3E Fig). In addition, PA-X deficient virus could only slightly increase the expression of these genes in control cells but not in TET2-KO cells (Fig 4G), suggesting TET2 is a key factor in regulating *STAT1* and ISG expression. TET2 could regulate the transcription of *STAT1*, *ISG15*, *ISG20*, and *IFIT5* through DNA demethylation.

### TET2 regulates STAT1 and ISG expression

**Fig 5:**
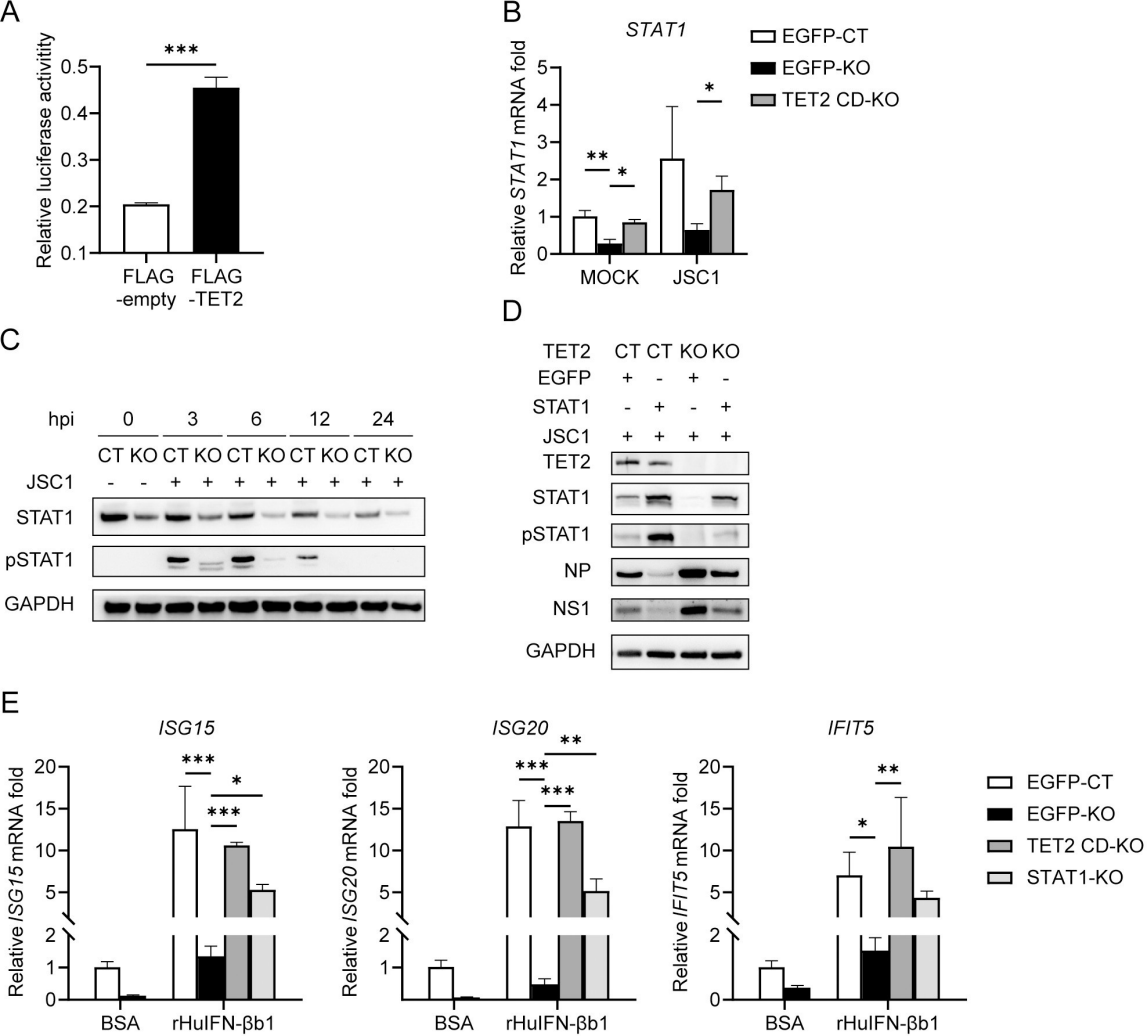
TET2 regulates STAT1 and ISG expression. (A) Dual-luciferase reporter assays were performed in HEK293T cells transfected with plasmids expression FLAG-TET2 and FLAG-empty proteins. Firefly luciferase activity driven by *STAT1* promoter was measured at 24 hpt and normalized to Renilla luciferase activity. Error bars were calculated from three technical replicates. (B) Control THP-1 cells ectopically expressing EGFP, and TET2-KO THP-1 cells ectopically expressing EGFP or FLAG-TET2 CD domain were infected with JSC1 at an MOI of 1, and *STAT1* mRNA levels were evaluated at 6 hpi via RT-qPCR. The data are expressed as fold changes relative to the mock EGFP-control group. (C) Control and TET2-KO THP-1 cells were infected with JSC1 at an MOI of 1 and harvested at 0, 3, 6, 12 and 24 hpi, followed by immunoblotting with indicated antibodies. (D) Control and TET2-KO THP-1 cells transduced with lentiviral vectors expressing EGFP or STAT1 were infected with JSC1 at an MOI of 1 and harvested at 6 hpi, followed by immunoblotting with indicated antibodies. (E) Control THP-1 cells ectopically expressing EGFP and TET2-KO THP-1 cells ectopically expressing EGFP, FLAG-TET2 CD domain or STAT1 were treated with 100 U/mL recombinant rHuIFN-βb1 and harvested at 3 hpi. The mRNA levels of *ISG15*, *ISG20* and *IFIT5* were evaluated via RT-qPCR. The data are expressed as fold changes relative to the mock EGFP-control group. Error bars represent ± SD for triplicate experiments. Statistical analysis was performed using Student’s t test or ANOVA method. *p < 0.05, **p < 0.01, ***p < 0.001. CT: control cells, KO: TET2 knockout cells.

STAT1 is the central mediator of the cellular response to IFNs and acts as an activator of ISG transcription in the nucleus during IAV infection [7, 8]. TET2 interacts with STAT1 to regulate the demethylation of CXCL10 and PD-L1 [53]. Thus, it is reasonable that TET2 interacts with STAT1 to regulate ISGs’ expression through demethylation, but whether TET2 could directly regulate *STAT1* expression remains elusive. To test it, we constructed plasmids expressing luciferase under the promoter of *STAT1* and co-transfected the plasmids with TET2-expression plasmids or empty vectors, followed by the luciferase activity measurement at 24 hpt. Significantly increased luciferase activity was observed in cells with TET2 overexpression (Fig 5A), which indicated that TET2 could directly regulate *STAT1* transcription. Consistently, the mRNA level of STAT1 was reduced by ~75% in TET2-KO THP-1 cells regardless of viral infection, and the reduction was counteracted by expressing the CD domain (Fig 5B). We further observed that the total STAT1 and the phosphorylated STAT1 (pSTAT1) proteins were significantly reduced in TET2 deficient cells regardless of IAV infection (Fig 5C). A similar reduction of STAT1 in the A549 cells with TET2 haploinsufficiency was observed (S2 Fig). Taken together, TET2 can regulate the expression of STAT1 by demethylating its promoter.

To further reveal the role of STAT1 in TET2-mediated anti-IAV immune response, we established STAT1 stable-overexpression in control and TET2-KO THP-1 cell lines. We found that overexpression of STAT1 can repress the increased viral protein expression due to TET2 deletion (Fig 5D). To exclude the influence of different levels of IFN expression in control and TET2-KO cells under viral infection, we treated cells with recombinant rHuIFN-βb1 to confirm the regulation of TET2 on ISGs further. After treatment with the same amount of recombinant rHuIFN-βb1 (100 U/mL) for 3 hours, the expression of *ISG15*, *ISG20*, and *IFIT5* was remarkably lower in TET2-KO cells than in control cells, and the expression can be restored by ectopic expression of either TET2 CD domain or STAT1 (Fig 5E). Notably, these results suggested that in the case of infection, the immune deficiency caused by TET2 dysfunction could be rescued by ectopically expressing either the CD domain of TET2 or STAT1. We demonstrated that TET2 regulates the expression of *STAT1* and ISGs, and STAT1 is a critical factor in response to the TET2 anti-IAV signal.

### IAV replication is enhanced in Tet2-deficient mice

**Fig 6:**
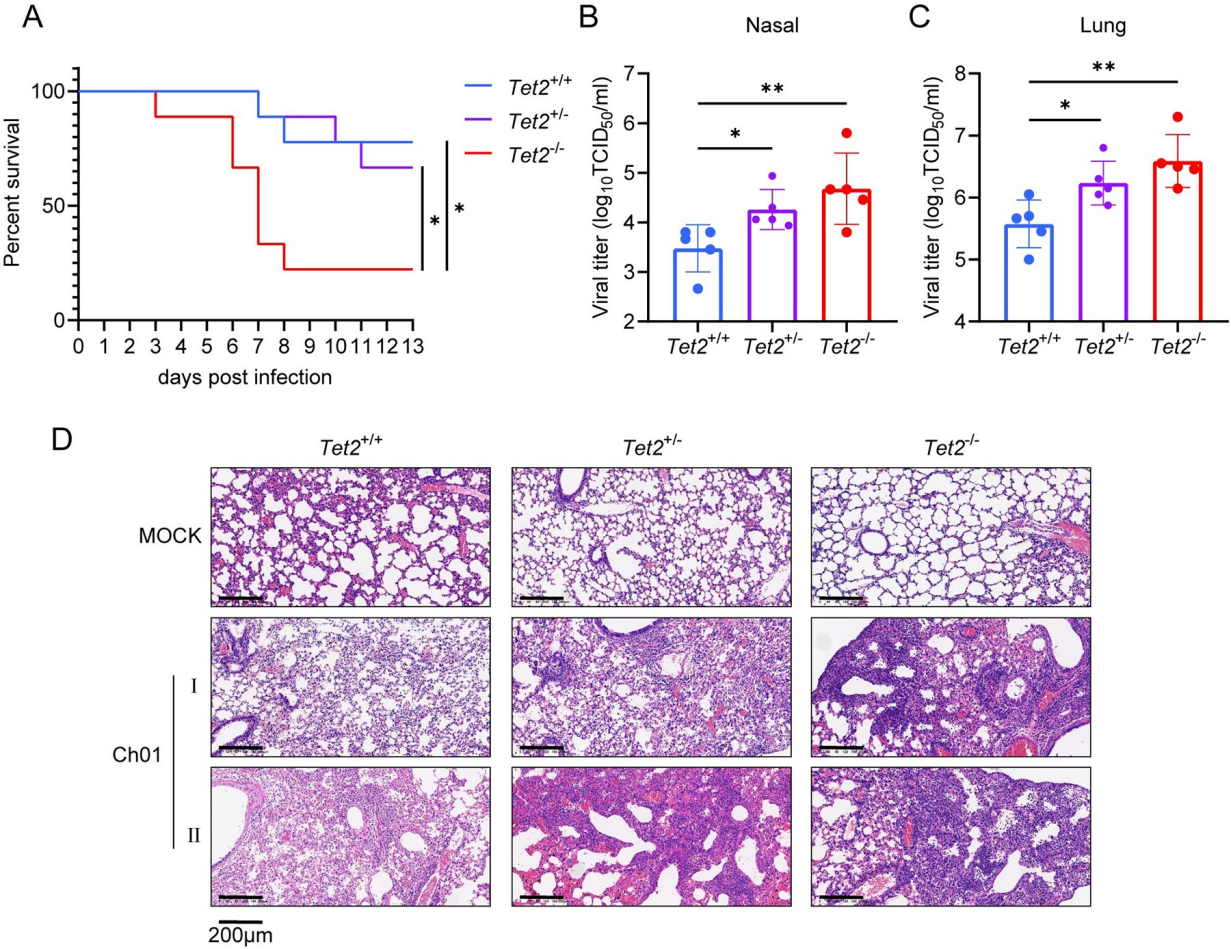
TET2 deletion enhances influenza infection and replication in mice. 4-week-old wild-type (*Tet2*^+/+^), heterozygous Tet2-KO (*Tet2*^+/-^) and homozygous Tet2-KO (*Tet2*^-/-^) C57BL/6 mice were intranasally infected with H9N2 Ch01 (10^4.5^ TCID_50_) and monitored every 24 h for mortality and morbidity. (A) Kaplan-Meier survival curves within 13 dpi are shown (n = 9/group). Statistical analysis was performed using log-rank (Mantel-Cox) test. *p < 0.05. Nasal lavage fluid (B) and lung grinding supernatant (C) of mice sacrificed on 4 dpi were collected for viral titration by TCID_50_ assay (n = 5/group). Error bars represent ± SD. Statistical analysis was performed using ANOVA method. *p < 0.05, **p < 0.01. (D) Histopathology of lungs of mice sacrificed on 7 dpi are shown (n = 1/group of mock treatment, n = 2/group of infected treatment). Scale bars, 200 μm.

To further confirm the anti-IAV activity of Tet2 *in vivo*, we infected wild-type (*Tet2*^+/+^), heterozygous Tet2-KO (*Tet2*^+/-^) and homozygous Tet2-KO (*Tet2*^-/-^) C57BL/6 mice with influenza virus A/Mink/China/01/2014(H9N2) (Ch01). Significantly lower survival rates were observed in the Tet2-KO mice within 13 days post-infection (dpi) (Fig 6A). We also detected the viral loads in both nasal lavage fluid and lung grinding supernatant of mice sacrificed on 4 dpi after Ch01 infection and found the viral loads increased over 10 times in the *Tet2*^-/-^ mice compared with *Tet2*^+/+^ mice (Fig 6B and 6C). The lung histological data showed that Tet2 deletion caused more severe lung pathologic changes on 7 dpi, with massive cell infiltration and obvious alveolar necrosis (Fig 6D). These data indicate that Tet2 contributes to host anti-IAV immunity, and Tet2 deletion enhances IAV replication, inducing more serious symptoms and pathological changes, and higher mortality.

## Discussion

Our research identified an unreported TET2 anti-IAV activity that can be inhibited by the IAV endoribonuclease PA-X. Specifically, PA-X degrades *TET2* mRNA dependent on its RNA endonuclease activity, leading to decreased TET2 expression in infected cells in a dose- and time-dependent manner. We further showed that loss of TET2 enhanced IAV replication, which was counteracted by ectopic expression of the TET2 CD domain or STAT1. Notably, we evidenced TET2 regulates the expression of STAT1 through DNA demethylation. Together, these results demonstrate that TET2 is a restriction factor of IAV.

Previous studies have identified many factors regulating TET2 expression, including transcription factors [54, 55], microRNAs and long noncoding RNAs [56–59]; and the four known protein degradation pathways, including caspase [60], calpeptin [61], proteasome [42] and autophagosome pathway [62], control TET2 protein levels. However, the study on the interaction of TET2 expression and virus infection is inadequate. Previous studies have found that TET2 expression can be downregulated by either transcript interaction during EBV infection [45] or protein degradation caused by HIV infection [42]. Our findings are similar: the TET2 protein level reduced upon IAV infection. However, the IAV-decreased TET2 protein level is mainly due to the reduced mRNA levels caused by the RNA endonuclease activity of viral PA-X protein.

Next, we confirmed that PA-X is crucial in IAV-regulated TET2 expression. Our results coincide with a study about IAV host shutoff activity in which the sequencing data showed TET2 transcriptions decreased by over 50% [18]. We showed that PA-X regulated TET2 expression by mRNA degradation instead of protein degradation. Using PA-X-deficient PR8 viruses, we further confirmed the role of PA-X in TET2 expression. However, we surprisingly found that TET2 transcription was induced by PA-X-deficient PR8 infection. IAV infection might stimulate TET2 transcription, but the transcripts were degraded by PA-X protein. Besides, the TET2 pre-mRNA levels slightly increased after H9N2 JSC1 infection. Previously studies have reported that TET2 could be induced by activated NF-κB p65 during inflammation [63, 64], and IAV infection could greatly activate the NF-κB pathway [5]. It would be interesting to study the activation of the transcription factor, such as p65, that leads to elevated TET2 expression during IAV infection in the future.

TET2 is a dioxygenase that catalyzes the conversion of 5mC to 5hmC [32–35]; thus, it widely activates gene expression by promoting DNA demethylation in their promoters. Using the hMeDIP-Seq technique, we discovered four IFN signaling pathway genes, *STAT1*, *ISG15*, *ISG20*, and *IFIT5*, which TET2 regulates through demethylation during IAV infection. ISG15, ISG20, and IFIT5 belong to the ISG family, acting as the host restriction factors to defense against IAV infection by disrupting viral proteins or targeting viral RNAs [12–16,65]. Hence, we inferred that TET2 could restrict IAV replication by demethylating *ISG15*, *ISG20*, and *IFIT5*. Wang et al. have reported that TET2 mediates the demethylation and expression of *IFITM3* genes, one of the ISGs, and plays a vital role in anti-HIV immune response [43]. However, there was no noticeable decrease of 5hmC level in *IFITM3* promoter in TET2-KO cells in our hMeDIP-Seq data, which may be due to different cells and viruses applied in the studies. Phosphorylated STAT1 activated by infection is responsible for IFN signal transduction and ISG expression activation [7,8], and TET2 has been reported to interact with STAT1 [53], which suggests that TET2 could also mediate demethylation of *ISG15*, *ISG20*, and *IFIT5* through interaction with STAT1. However, it was unclear whether TET2 could regulate STAT1 expression, and little is known about the detailed regulation of STAT1 expression by IAV. Our study revealed that the TET2-mediated DNA demethylation was essential for STAT1 expression. Notably, although supplementation of rHuIFN-βb1 couldn’t impair the elevated viral replication in TET2-KO cells, ectopic expression of STAT1 can partially restore the immune function due to TET2 deletion, which indicated that STAT1 plays a crucial role in TET2-mediated anti-IAV immunity.

PA-X has been reported to contribute to the virulence of IAV, but PA-X deficiency results in different consequences of viral replication of varying IAV strains [66]. Xu et al. showed that loss of PA-X in the H1N2 strain decreased viral replication in pig cells and pigs [67], and Hayashi T et al. demonstrated that PA-X of pandemic H1N1 contributes to viral growth and suppression of the host anti-viral responses [30]. We also noticed that PA-X deficiency in H1N1 PR8 could slightly impair viral replication and promote *STAT1* and ISG transcription. Indeed, we cannot exclude the non-specific endonuclease activity of PA-X on STAT1 and ISG expression. However, wild-type and PA-X-deficient viruses showed a greatly increased viral protein expression in TET2 knockout cells. And the expression of *STAT1* and ISGs in the wild-type and PA-X-deficient virus infected TET2 knockout cells was extremely decreased. Notably, the deduction of *STAT1* and ISG expression by wild-type and PA-X-deficient viruses were comparable. These results indicate TET2 is a crucial factor regulating STAT1 and ISG expression.

Furthermore, we verified the anti-IAV activity of Tet2 *in vivo*. We found that Tet2 contributes to host anti-IAV immunity, and Tet2 deletion results in higher viral load and mortality, as well as more severe symptoms and pathological changes, supporting the conclusion from the *in vitro* experiments that TET2 restricts IAV replication. However, it is remarkable that TET2 plays multiple roles in immunity *in vivo*. TET2 regulates the expression of cytokines, such as Il-6, Il-1β, and Cxcl10, produced mainly in the inflammatory resolution stage [40,41,63,64], contributing to the recruitment and activation of immune cells. Besides, TET2 promotes cytokine-induced myelopoiesis upon infection by decreasing 5mCs in *Socs3* mRNA, thereby activating the emergency production of mature innate immune cells during pathogen infection [39]. Moreover, TET2 regulates the differentiation and proliferation of mast cells [68], T cells [69,70] and B cells [71–73] during infection. For instance, Tet2 promotes DNA demethylation and activation of cytokine genes, including Il-10, Ifn-γ, and Il-17 expression in T cells, which plays a crucial role in Tr1 cell development [69]. Tet2 regulates the differentiation of GC B cells into plasma cells that secrete substantial antibodies, including lgG and lgλ [72]. Besides regulating IFN signaling in innate immunity, TET2 also participates in the resolution of inflammation and the activation of adaptive immunity, which are also important for the host to defend against IAV infection. Thus, it is worth determining the exact role of TET2 in anti-IAV infection and pathogenesis *in vivo* in the future.

## Materials and methods

### Ethics statement

All experiments were conducted in compliance with a protocol approved by the Experimental Animal Ethics Committee of Zhejiang University (protocol No.: 23426).

### Cell culture and viruses

Human myeloid leukemia mononuclear (THP-1) cells, human alveolar adenocarcinoma epithelial (A549) cells, human embryonic kidney (HEK293T) cells, and Madin-Darby canine kidney (MDCK) cells were used in this study. THP-1 cells, A549 cells, HEK293T cells, and MDCK cells were cultured with Roswell Park Memorial Institute (RPMI)-1640, Ham’s F-12 nutrient medium (F-12), Dulbecco’s modified Eagle’s medium (DMEM), and minimum essential medium (MEM), respectively. These media were purchased from Thermo Fisher (Waltham, MA, USA), and were supplemented with 10% fetal bovine serum (FBS) (ExCell Biology, Shanghai, China) and 1% Penicillin-Streptomycin antibiotics. All cells were maintained in a humidified incubator containing 5% CO_2_ at 37°C.

The influenza virus A/swine/Jiangsu/C1/2008 (H9N2) (JSC1), A/mink/China/01/2014 (H9N2) (Ch01), A/Puerto Rico/8/34 (H1N1) (PR8) and A/Zhejiang/163/2020 (H3N2) (ZJ163) were propagated in 10-day-old embryonated chicken eggs. Viral titers were evaluated by calculating the 50% tissue culture infectious (TCID_50_) per milliliter using the Reed and Muench method in MDCK cells.

### Antibodies and reagents

Primary antibodies in this study were anti-TET2 rabbit polyclonal antibody (catalogue No. 21207-1-AP, Proteintech, Chicago, IL, USA), anti-IAV NS1 mouse monoclonal antibody (catalogue No. sc-130568, Santa Cruz, Dallas, TX, USA), anti-IAV NP mouse monoclonal antibody (catalogue No. ab128193, Abcam, England, UK), anti-STAT1 rabbit polyclonal antibody (catalogue No. 10144–2-AP, Proteintech, Chicago, IL, USA), anti-pSTAT1 (Tyr701) rabbit polyclonal antibody (catalogue No. 7649, Cell Signaling, Danvers, MA, USA), anti-GAPDH mouse monoclonal antibody (catalogue No. FD0063, FD Bio, Hangzhou, China), anti-FLAG mouse monoclonal antibody (catalogue No. HT201-01, TransGen Biotech, Beijing, Ching), anti-5hmC rabbit polyclonal antibody (catalogue No. 39791, Active Motif, Carlsbad, CA, USA). Secondary antibodies were purchased from FD Bio (Hangzhou, China), including horseradish peroxidase (HRP)-conjugated goat anti-rabbit IgG(H+L) (catalogue No. FDR007) and HRP-conjugated goat anti-mouse IgG(H+L) (catalogue No. FDM007). All antibodies were used at recommended dilutions according to the manufacturer’s protocols.

Recombinant Human Interferon-beta1b (rHuIFN-β1b) (106–05) was obtained from PrimeGene (Shanghai, China), reconstituted in sterile distilled water containing 0.1% BSA-V (Sigma, St. Louis, MO, USA). Doxycycline (ST039A) was obtained from Beyotime (Shanghai, China), and dissolved in sterile distilled water. Protein degradation inhibitors MG132 (HY-13259), Z-VAD-FMK (HY-16658B), and Calpeptin (HY-100223) were obtained from MCE (Monmouth Junction, NJ, USA), dissolved in dimethyl sulfoxide (DMSO) (Sigma, St. Louis, MO, USA) according to the manufacturer’s protocols. NH_4_Cl was obtained from Sinopharm Chemical Reagent Co., Ltd. (Shanghai, China), and dissolved in sterile distilled water.

### Plasmids and transfection

The reverse genetic plasmids encoding eight segments of PR8 were constructed previously. The human *TET2* (NCBI reference number: NP_001120680.1) and *STAT1* gene (NCBI reference number: NP_001371809.1), as well as the viral PA (NCBI reference number: AOW32249.1) and NS1 (NCBI reference number: AOW32255.1) gene of JSC1 were obtained from JSC1-infected THP-1 cells by RT-PCR. EGFP sequences were amplified from the eukaryotic expression vector pIRES2-EGFP vector by PCR. PA-X was generated by overlapping 1–570 nucleotides (nt) with 572–760 nt of PA nucleotide sequence via PCR. DNAs of the TET2 CD domain and PA-N were truncated fragments of TET2 (1073–2002 aa) and PA/PA-X (1–191 aa), respectively, and were amplified by PCR approach. Point mutations in PA (L201Stop) and PA-X/PA-N (D108A) were generated by PCR mutagenesis and verified by DNA sequencing. For transient transfection in HEK293T cells, TET2, PA, PA L201Stop, PA-X, PA-X D108A, and PA-N D108A were subcloned to eukaryotic expression vector pCMV-FLAG at *Hin*dIII and *Xho*I sites, respectively, and NS1 with an MYC tag at the N terminus was inserted into pIRES2-EGFP at *Xho*I and *Bam*HI sites. For the generation of stable expression cell lines, an N-terminal FLAG tag was added to PA-X, PA-X D108A, and TET2 CD. Then, EGFP, FLAG-PA-X, and FLAG-PA-X D108A were subcloned to doxycycline (DOX)-inducible expression vector pRetroX-TetOne-puro at *Eco*RI and *Bam*HI sites, respectively; EGFP, FLAG-TET2 CD, and STAT1 were inserted into the backbone of lentiviral vector lentiCas9-blast after Cas9 excision with *Xba*I and *Bam*HI. For the generation of PA-X-deficient PR8, point mutations in PR8 PA (L201Stop) were generated by PCR mutagenesis, and then PR8 PA (L201Stop) was constructed in reverse genetic plasmids pBZ61A18 at *Pst*I sites. For the generation of TET2-KO cells, the gRNA sequence of TET2 (GATTCCGCTTGGTGAAAACG) [42] was constructed in CRISPR-Cas9 lentiviral vector lentiCRISPRv2-neo at *Bsm*BI sites. For dual-luciferase reporter assay, PCR-amplified STAT1 promoter region (chr2:191,878,977–191,990,955) were subcloned to luciferase reporter vector pGL3-basic at *Xho*I and *Bam*HI sites.

Transfection of plasmids to HEK293T cells was performed using transfection reagent lipofectamine 2000 (Thermo Fisher, Waltham, MA, USA) following manufacturer’s instructions. Briefly, 2.5 μg total DNA and 7.5 μL lipofectamine 2000 were diluted in 150 μL Opti-MEM medium (Thermo Fisher, Waltham, MA, USA) respectively, then mixed and incubated at room temperature for 10 min. The mixture was added to each well containing 70%-90% confluent HEK293T cells plated one day before transfection in a 6-well plate. Then the medium was changed after 4 hours, and cells were incubated for another 24 hours.

### Generation of stable expression cell lines and TET2-KO cells

Retrovirus expressing EGFP, FLAG-PA-X, or FLAG-PA-X D108A were packaged by 1.5 μg constructed retrovirus vector, 0.5 μg pVSV-G, and 0.5 μg pMD-MLV in HEK293T cells. Lentiviruses for CRISPR and lentiviruses expressing EGFP, FLAG-TET2 CD or STAT1 were produced by transfection in HEK293T cells using 1 μg constructed lentiviral vector, 1 μg psPAX2 and 0.5 μg pMD2G. All retroviruses and lentiviruses were tittered on HEK293T cells. Transductions of both THP-1 cells and A549 cells were performed with an MOI of 0.5 for three days. The cells were selected with 1.5 μg /mL puromycin (InvivoGen, San Diego, CA, USA), 2 μg /mL blasticidin (InvivoGen, San Diego, CA, USA), or 500 μg/mL geneticin G418 (Thermo Fisher, Waltham, MA, USA) for 7 days. For doxycycline-inducible expression, cells were treated with 0.2 mg/mL doxycycline for 24 hours to induce EGFP, FLAG-PA-X, and FLAG-PA-X D108A expression before RNA or protein sample collection. RT-qPCR and Western Blot were used to evaluate the ectopic expression. For single clones of TET2-KO cells isolation, the antibiotic-resistant pool of knockout cells was limiting diluted to 0.5 cells per well in 96-well plates. The recovered KO clones were validated by DNA sequencing and Western Blot.

### Viral infection and TCID_50_ assay

IAV infection experiments were conducted using viral growth medium consisting of serum-free medium supplemented with 2% BSA-V and 2 μg/mL of tosylsulfonyl phenylalanyl chloromethyl ketone (TPCK)-trypsin (Sigma, St. Louis, MO, USA). After the cell culture medium was withdrawn, 1x10^6^ THP-1 cells or 90% confluent A549 cells plated one day before infection were washed twice with phosphate-buffered saline (PBS) (Thermo Fisher, Waltham, MA, USA). Then viruses in viral growth medium were added to the cells at MOIs indicated in the Figure legends. After adsorption at 37°C for 1 hour, the inoculum was removed and replaced with a fresh viral growth medium. At the indicated time points post-infection, cells were collected to prepare RNA and protein samples, and viral supernatants were harvested for TCID_50_ assay.

For viral titer measurement, 8x10^3^ per well MDCK cells were plated in 96-well plates, grew to 90%–95% confluence overnight, and were washed twice with PBS before inoculation. The supernatants were diluted serially in MEM supplemented with 2% BSA-V and 2 μg/mL of TPCK-trypsin, and then inoculated into the cells. The IAV-induced cytopathic effect (CPE) was monitored for 24–96 hours. TCID_50_ was then calculated by the Reed and Muench formula.

### Generation of recombinant viruses by reverse genetic system

HEK293T and MDCK cells prepared for transfection were seeded at a ratio of 7:1. 0.8 μg of each reverse genetic plasmids encoding eight segments of PR8 were transfected into the cells. The medium was changed into viral growth medium after 4 hours, and cells were incubated for another 48 hours. Transfer supernatants into MDCK cells for amplification. Rescued viruses were detected using hemagglutination assays. Viral RNA was extracted for RT-PCR and sequencing. Viral titers were detected by TCID_50_ assays.

### Western Blot assay

Cells were collected and washed twice with PBS, then lysed with RIPA lysis buffer (Beyotime, Shanghai, China) containing a protease inhibitor cocktail (Roche, Basel, Switzerland). The cell lysates were centrifuged at 12,000 × g for 10 min at 4°C, and the supernatants were collected. Then, the supernatants were added with SDS-PAGE sample loading buffer (FD Bio, Hangzhou, China) and boiled at 100°C for 10 min. Equal amounts of protein samples were subjected to SDS-polyacrylamide gel (FD Bio, Hangzhou, China) electrophoresis and then transferred to a PVDF membrane (Millipore, Darmstadt, Germany). The membrane was probed with indicated primary and related secondary antibodies and finally filmed using the ECL detection reagents (CYANAGEN, Bologna, Italy).

### Reverse transcription and quantitative real-time PCR (RT-qPCR)

A two-step RT-qPCR was used to examine specific mRNA levels. Total RNA was extracted and purified according to the manufacturer’s instructions (Easy-do Bio, Zhejiang, China). Reverse transcription was performed using HiScript III RT SuperMix for qPCR (+gDNA wiper) (Vazyme, Nanjing, China). qPCR was carried out with ChamQ Universal SYBR qPCR Master Mix (Vazyme, Nanjing, China), on the Stratagene Mx3005P qPCR Instrument. The primers used are listed in S1 Table. The Mean threshold cycle (Ct) values of each gene was calculated. The housekeeping gene *GAPDH* or *18S rRNA* was used for normalization in gene expression analysis. Relative fold changes in gene expression among groups were determined using the 2^−ΔΔCt^ method.

### Dual-luciferase reporter assay

HEK293T cells in a 24-well plate were transfected with pCMV-FLAG-TET2 constructs or pCMV-FLAG-empty as a control group (0.5 μg each), the construct pGL3-STAT1-Luc (0.1 μg) and internal control pRL-TK (50 ng; Promega), by using 2 μL lipofectamine 2000. Then the medium was changed after 4 hours, and cells were incubated for another 24 hours. Cell lysates were prepared using the dual-luciferase assay kit (Vazyme, Nanjing, China) according to the manufacturer’s instructions. The luciferase activities were measured on a SYNERGY HTX Multi-Mode Reader (BioTek, VT, USA).

### RNA immunoprecipitation (RIP)

RIP was performed with anti-FLAG M2 beads (M8823, Sigma, St. Louis, MO, USA) using cell lysate of HEK293T cells transfected with pCMV-FLAG-PA-X D108A, pCMV-FLAG-PA-N D108A or pCMV-FLAG-empty for 24 hours (see above). Briefly, cells were lysed with RIPA lysis buffer containing a protease inhibitor cocktail and RNase inhibitor (Beyotime, Shanghai, China), and the supernatants were collected after centrifugation. Ten percent of the cell lysate supernatant was taken as an input sample. Then, the rest of the supernatant was added with anti-FLAG M2 beads and incubated with rotation overnight at 4°C according to the manufacturer’s protocols. Beads were collected using a magnetic separator and washed five times with RIP buffer (50 mM Tris-HCl pH 7.5, 250 mM KCl, 5 mM EDTA, 1% NP-40, 0.5 mM DTT, and 200 units/mL RNase inhibitor). After the fifth wash, the beads were resuspended with 200 μl of proteinase K digestion buffer (50 mM Tris-HCl pH 8, 10 mM EDTA, 200 mM NaCl, 0.5% SDS, 200 μg/mL proteinase K) and then were incubated with rotation for 30 minutes at 55°C. RNA purification of the immunoprecipitated sample (IP sample) and input sample, as well as RT-qPCR assay, were performed as mentioned above. The recovery efficiency (percentage of the input) of the IP RNA was calculated as 2^*CT* IP—*CT* input^.

### Hydroxymethylated DNA immunoprecipitation (hMeDIP)

hMeDIP was performed in control and TET2-KO THP-1 cells infected with JSC1 at an MOI of either 3 for sequencing or 1 for qPCR. Cells were collected at 6 hpi, and genome DNA was extracted and purified using Trelief Animal Genomic DNA Kit according to the manufacturer’s instructions (Tsingke Biotechnology, Beijing, China). DNA was sheared into approximately 200–1,000 bp fragments by sonication using a Branson sonifier (10 sec on, 10 sec off, 30% powder; 3 min at 4°C). After the denaturation of DNA, 10% of the denatured DNA was taken as an “input” sample. The remaining denatured DNA was mixed with 10 × IP buffer (100 mM Na-phosphate pH 7.0 mono-dibasic, 1.4 M NaCl, 0.5% Triton X-100) and 1 μg of α-5hmC antibody (ActiveMotif, CA, USA), and incubated with rotation overnight at 4°C. The DNA-antibody mixture was transferred to the Dynabeads Protein G (Thermo Fisher, Waltham, MA, USA) and incubated for 2 hours with rotation at 4°C. Beads were collected using a magnetic separator and washed five times with 1 × IP buffer. After washing, beads were resuspended with 200 μL of proteinase K digestion buffer and incubated with rotation for 3 hours at 55°C. DNA fragments of the immunoprecipitated (IP) and input sample were purified using QIAquick PCR kit (QIAGEN, Shanghai, China) according to the manufacturer’s protocols. The qPCR assay was performed as mentioned above. The 5hmC enrichment (percentage of the input) was calculated as 2^*CT* IP—*CT* input^. For sequencing, DNA fragments were amplified with Illumina 8-bp dual index primers and sequenced by the Illumina HiSeq 2000 platform at Active Motif (https://www.activemotif.com/catalog/834/hmedip-sequencing-service) (Shanghai, China). Briefly, base calls were performed using bcl2fastq v2.17 for Novaseq output. hMeDIP-Seq reads were trimmed from 3’ end until the final base had a quality score > 30, using Trimmomatic v0.36, discarding reads left with < 36 bp, and were aligned to the UCSC hg19 genome using BWA-0.7.17. Peaks between IP sample and input sample was called by MACS-1.4.2 with the significance cut-off *p*-value < = 1e-5.

### Animal experiments

*Tet2*^-/-^ mice in the C57BL/6 background were generously provided by Dr. Xing Chang from Westlake University [73]. Wild-type C57BL/6 mice were purchased from the SLAC Laboratory Animal Co. (Shanghai, China). *Tet2*^-/-^ mice and wild-type C57BL/6 mice were bred to generate *Tet2*^+/-^ mice. The *Tet2*^+/-^ mice were bred together to get *Tet2*^+/+^, *Tet2*^+/-^ and *Tet2*^-/-^ mice for experiments. Four-week-old mice were lightly anesthetized with isoflurane and intranasally infected with 10^4.5^ TCID_50_ H9N2 Ch01 in 20 μL diluent or 20 μL PBS as a negative control. Nine mice per group were monitored for mortality every 24 hours, and then draw survival curves using the Kaplan-Meier method; five mice per group were euthanized on 4 dpi, and nasal lavage fluid and lung sample were collected for virus titration by TCID_50_ assay; two infected mice and one mock mouse per group were euthanized on 7 dpi, and lungs were collected and fixed with 4% paraformaldehyde (Sinopharm Chemical Reagent Co., Ltd., Shanghai, China) and embedded in paraffin. 4-μm-thickness tissue sections were sliced and stained with hematoxylin and eosin (Sigma, St. Louis, MO, USA) and subsequently were dehydrated and mounted for digital scanning using KF-PRO Digital Slide Scanner (Konfoong Bioinformation Tech Co., Ltd., Ningbo, China).

### Statistical analysis

Quantitative data were summarized and visualized with GraphPad Prism software as mean ± standard deviation (SD) from at least three independent experiments, unless otherwise indicated in the Figure legends. Normally, statistical analysis between two groups was performed with paired two-tailed Student’s t test, among multiple groups was performed with one-way ANOVA method, and multiple comparisons were carried out by two-way ANOVA method considering cell type and treatment as factors; a log-rank (Mantel-Cox) test was used for the mouse survival curve analysis. Levels of significance were indicated as *p < 0.05, **p < 0.01, ***p < 0.001.

## Supporting information

S1 Fig PA-X degrade *TET2* mRNA dependence on the endoribonuclease activity. **Related to Fig 2.** (A) HEK293T cells transfected with indicated plasmids and then treated with inhibitors of 26S proteasome (MG132, 10 mM), calpain (Calpeptin, 50 mM), caspase (Z-VAD-FMK, 100 mM), or lysosome (NH_4_Cl, 20 mM) for 24 hours, followed by immunoblotting. (B) RNA and protein samples were collected from A549 cells expressing doxycycline-inducible FLAG-PA-X, FLAG-PA-X D108A, or EGFP 18 hours after the addition of doxycycline, followed by immunoblotting analysis and RT-qPCR detection. The RT-qPCR data are expressed as fold changes relative to the EGFP control. Statistical analysis was performed using ANOVA method. **p < 0.01. (C) Diagrams of expressing proteins in indicated viral PA segment are shown. (D) Virus growth curves of PR8 WT and PR8 ΔPA-X in MDCK cells over 72 h. MDCK cells were infected with either PR8 WT or PR8 ΔPA-X at an MOI of 0.01, and the culture supernatants were collected at 24, 36, 48 and 72 hpi for viral titration by TCID_50_ assay. Statistical analysis was performed using Student’s t test. *p < 0.05.
(TIF)

S2 Fig TET2 haploinsufficiency enhances IAV replication and reduces STAT1 expression in A549 cells. **Related to Fig 3 and Fig 5.** Non-targeting gRNA control (CT) and heterozygous TET2-KO A549 cells established by CRISPR-Cas9 system were infected with JSC1 at an MOI of 1 and harvested at 0, 6, 12 and 24 hpi, followed by immunoblotting with indicated antibodies.
(TIF)

S3 Fig TET2 haploinsufficiency attenuates IAV-induced IFNβ and ISG expression in A549 cells. **Related to Fig 4.** (A-E) Non-targeting gRNA control (CT) and heterozygous TET2-KO A549 cells were infected with JSC1 at an MOI of 1 and harvested at 12 hpi. mRNA levels of *IFNB1* (A), *STAT1* (B), *ISG15* (C), *ISG20* (D) and *IFIT5* (E) were evaluated via RT-qPCR. The data are expressed as fold changes relative to the mock CT group. Error bars represent ± SD for triplicate experiments. Statistical analysis was performed using ANOVA method. *p < 0.05, **p < 0.01, ***p < 0.001.
(TIF)

S1 Table Primer sequences used in quantitative real-time PCR. (DOCX)
