# Supplementary figures and images for: Influenza A virus inhibits TET2 expression by endoribonuclease PA-X to attenuate type I interferon signaling and promote viral replication
Source: PLoS Pathog. 2023 Jul 27;19(7):e1011550. doi: 10.1371/journal.ppat.1011550 (PMC10409264; doi:10.1371/journal.ppat.1011550)

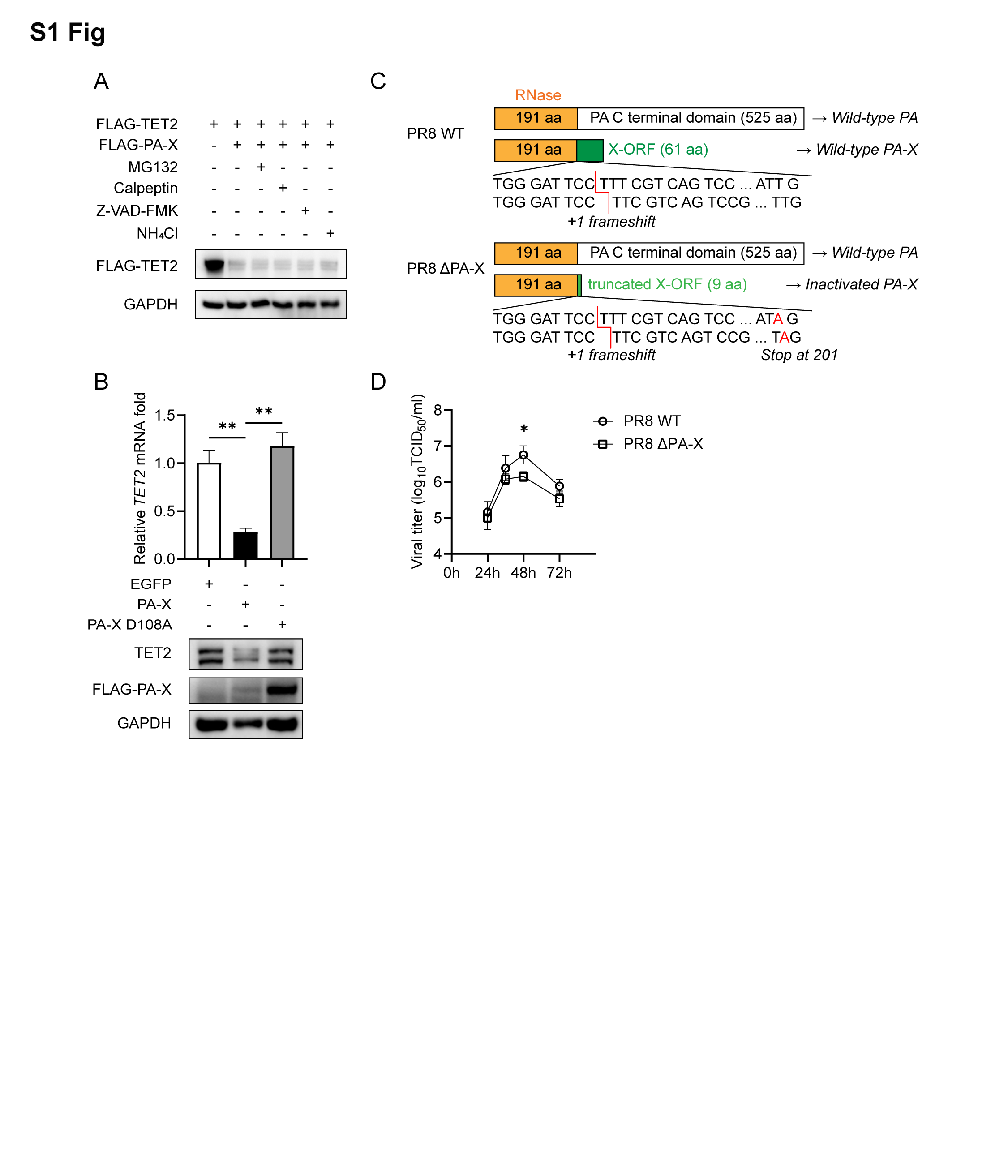

Supplement: S1 Fig — Related to Fig 2. (A) HEK293T cells transfected with indicated plasmids and then treated with inhibitors of 26S proteasome (MG132, 10 mM), calpain (Calpeptin, 50 mM), caspase (Z-VAD-FMK, 100 mM), or lysosome (NH4Cl, 20 mM) for 24 hours, followed by immunoblotting. (B) RNA and protein samples were collected from A549 cells expressing doxycycline-inducible FLAG-PA-X, FLAG-PA-X D108A, or EGFP 18 hours after the addition of doxycycline, followed by immunoblotting analysis and RT-qPCR detection. The RT-qPCR data are expressed as fold changes relative to the EGFP control. Statistical analysis was performed using ANOVA method. **p < 0.01. (C) Diagrams of expressing proteins in indicated viral PA segment are shown. (D) Virus growth curves of PR8 WT and PR8 ΔPA-X in MDCK cells over 72 h. MDCK cells were infected with either PR8 WT or PR8 ΔPA-X at an MOI of 0.01, and the culture supernatants were collected at 24, 36, 48 and 72 hpi for viral titration by TCID50 assay. Statistical analysis was performed using Student’s t test. *p < 0.05. (TIF) [file ppat.1011550.s001.tif]

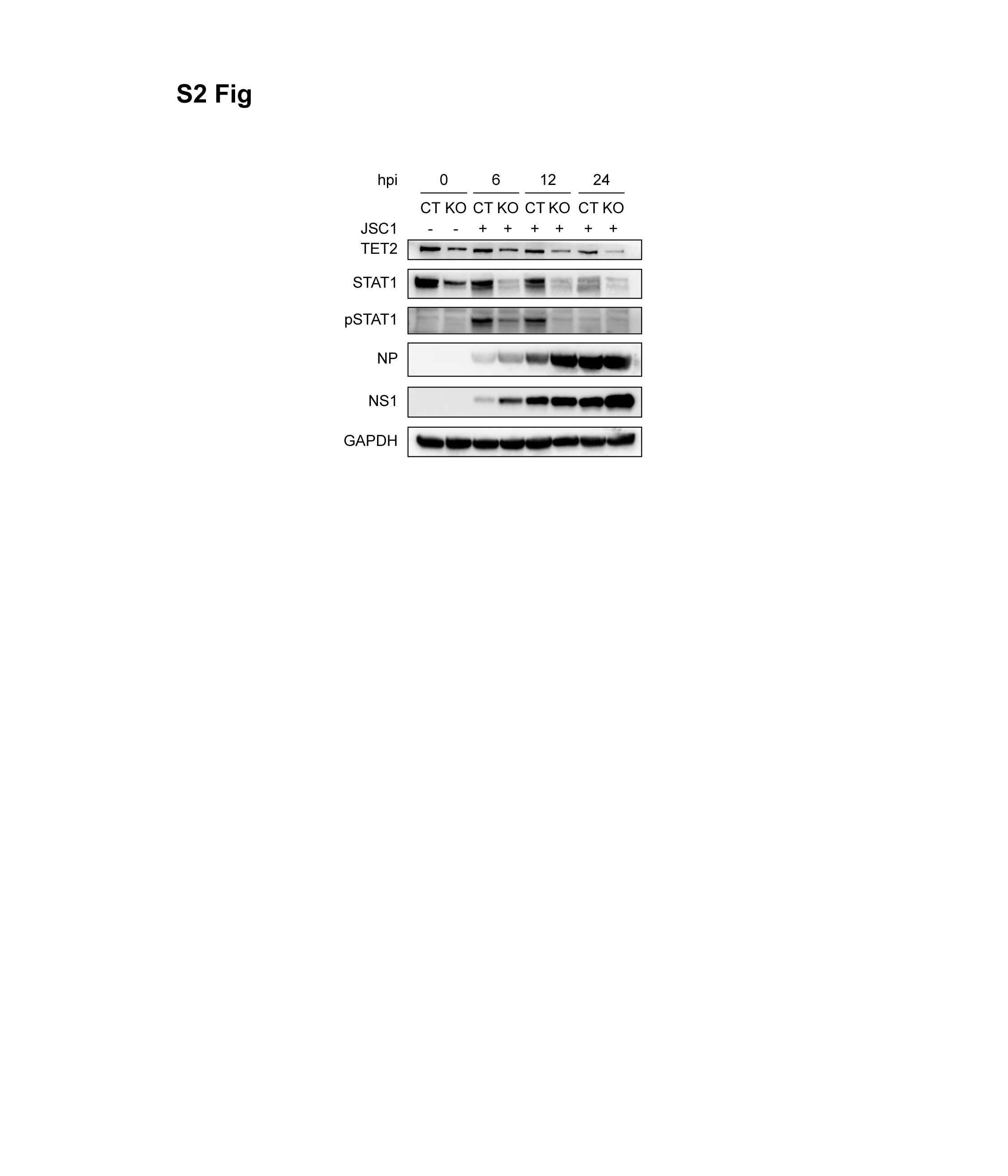

Supplement: S2 Fig — Related to Fig 3 and Fig 5. Non-targeting gRNA control (CT) and heterozygous TET2-KO A549 cells established by CRISPR-Cas9 system were infected with JSC1 at an MOI of 1 and harvested at 0, 6, 12 and 24 hpi, followed by immunoblotting with indicated antibodies. (TIF) [file ppat.1011550.s002.tif]

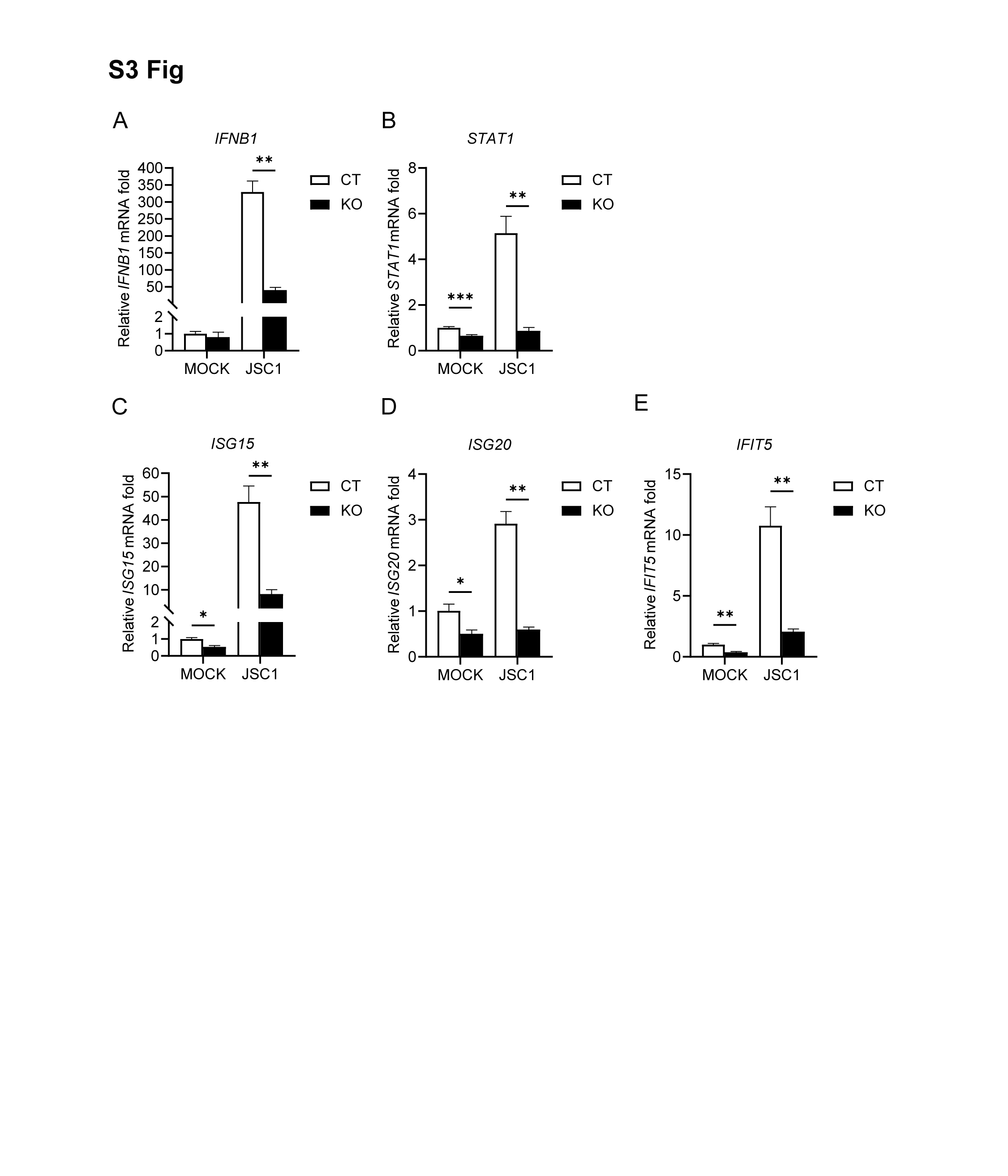

Supplement: S3 Fig — Related to Fig 4. (A-E) Non-targeting gRNA control (CT) and heterozygous TET2-KO A549 cells were infected with JSC1 at an MOI of 1 and harvested at 12 hpi. mRNA levels of IFNB1 (A), STAT1 (B), ISG15 (C), ISG20 (D) and IFIT5 (E) were evaluated via RT-qPCR. The data are expressed as fold changes relative to the mock CT group. Error bars represent ± SD for triplicate experiments. Statistical analysis was performed using ANOVA method. *p < 0.05, **p < 0.01, ***p < 0.001. (TIF) [file ppat.1011550.s003.tif]
