# Supplementary material for: Influenza A virus inhibits TET2 expression by endoribonuclease PA-X to attenuate type I interferon signaling and promote viral replication
Source: PLoS Pathog. 2023 Jul 27;19(7):e1011550. doi: 10.1371/journal.ppat.1011550 (PMC10409264; doi:10.1371/journal.ppat.1011550)
Supplement: S1 Table — (DOCX) [file ppat.1011550.s004.docx]

**Table S1.** List of primers for qRT-PCR assay in this study.

| Target gene | Direction | Sequence (5′–3′) |
| --- | --- | --- |
| *TET2* | Forward | GATAGAACCAACCATGTTGAGGG |
|  | Reverse | TGGAGCTTTGTAGCCAGAGGT |
| pre-*TET2* | Forward | ctttgggacctgtagttgaggc |
|  | Reverse | gaacgtgaagctgctcatcctc |
| *NS1* | Forward | TCGAAACAGCTACTCGTGCG |
|  | Reverse | ACTGTGAAGCAGGCACAGAA |
| *RIG-I* | Forward | ACGCAGCCTGCAAGCCTTCC |
|  | Reverse | TGTGGCAGCCTCCATTGGGC |
| *TRL3* | Forward | caaacacaagcattcggaatctg |
|  | Reverse | aaggaatcgttaccaaccacatt |
| *TLR7* | Forward | tcgtggactgcacagacaag |
|  | Reverse | ggtatgtggttaatggtgagggt |
| *IRF3* | Forward | GCAGGAGGATTTCGGAATCTTC |
|  | Reverse | GGAAATTCCTCTTCCAGGTTGG |
| *IRF7* | Forward | ggaggcccaaggagaagag |
|  | Reverse | tgctgctatccagggaagac |
| *IFNB1* | Forward | TTGTTGAGAACCTCCTGGCT |
|  | Reverse | TGACTATGGTCCAGGCACAG |
| *STAT1* | Forward | TGGCCCTAAAGGAACTGGAT |
|  | Reverse | CACTATCCGAGACACCTCGTC |
| *ISG15* | Forward | CGCAGATCACCCAGAAGATCG |
|  | Reverse | CGCAGATCACCCAGAAGATCG |
| *ISG20* | Forward | TGCTGTGCTGTACGACAAGT |
|  | Reverse | GGAAGTCGTGCTTCAGGTCA |
| *OAS1* | Forward | tgtccaaggtggtaaagggtg |
|  | Reverse | ccggcgatttaactgatcctg |
| *IFITM3* | Forward | caaggaggagcacgagg |
|  | Reverse | ttgaacagggaccagacg |
| *IFIT5* | Forward | acaagttggagtgtcctgaga |
|  | Reverse | aagccgctttagccttttgata |
| *MOV10* | Forward | gggccagtgtttcgagagttt |
|  | Reverse | tcttggtgacgtaggccaga |
| *TRIM25* | Forward | AATCGGCTGCGGGAATTTTTC |
|  | Reverse | TCTCACATCATCCAGTGCTCT |
| *18S rRNA* | Forward | cgttcagccacccgagattg |
|  | Reverse | ctgggaattcctcgttcatgg |
| *GAPDH* | Forward | tggccttccgtgttcctac |
|  | Reverse | gagttgctgttgaagtcgca |
| hMeDIP qPCR primer of *STAT1* | Forward | ggaacagccgcgtctaattg |
|  | Reverse | GAAAGCGAAACTACCCGGCA |
| hMeDIP qPCR primer of *ISG15* | Forward | cctgtggtcccagctacgt |
|  | Reverse | ctggagtgcagaggcacga |
| hMeDIP qPCR primer of *ISG20* | Forward | ctgcatgtaagtggacctgtg |
|  | Reverse | cacaagggacacgctctcc |
| hMeDIP qPCR primer of *IFIT5* | Forward | cgctgccatcatgaggtaag |
|  | Reverse | cctgaaaaggccgcagaact |
